# Influence of ecological characteristics and phylogeny on native plant species' commercial availability

**DOI:** 10.1002/eap.3070

**Published:** 2024-12-18

**Authors:** Jack Zinnen, Rebecca S. Barak, Jeffrey W. Matthews

**Affiliations:** ^1^ Department of Natural Resources and Environmental Sciences University of Illinois Urbana‐Champaign Urbana Illinois USA; ^2^ Negaunee Institute for Plant Conservation Science and Action Chicago Botanic Garden Glencoe Illinois USA; ^3^ Program in Plant Biology and Conservation Northwestern University Evanston Illinois USA; ^4^ Present address: Illinois Natural History Survey, Prairie Research Institute University of Illinois Urbana‐Champaign Champaign Illinois USA

**Keywords:** biodiversity, ecological conservatism, native plant nursery, phenology, plant trade, plant vendors, restoration, specialization

## Abstract

Plant vendors generate a commercial species pool, the subset of species in a regional flora that is purchasable. The availability of plant species from commercial vendors can influence the composition and outcomes of conservation, landscaping, and restoration plantings. Although previous research suggests that most plant species are unavailable, there is little information that identifies the plant characteristics associated with commercial availability. We studied the composition of the commercial species pool by examining the ecology, phylogeny, and phenology of a regional flora in the Midwestern United States. We used a database of native plant species sold by 557 vendors throughout the Midwest (USA) to characterize species' availability. We compiled ecological characteristics of all plant species, including range size, growth form, moisture requirements, and conservatism—meaning fidelity to high‐quality natural areas. We characterized phenological (bloom time) data for a subset of the regional flora. Finally, we constructed a regional phylogeny to assess the phylogenetic signal of plant availability. We expected that commercially unavailable species would be niche specialists or short‐lived (often nonconservative “weedy”) species, and that they would bloom earlier in the season and for a shorter time. We found that commercially available species were more long‐lived, had larger range sizes, had intermediate fidelities to wetlands and high‐quality or disturbed natural areas, and were associated with certain plant types, especially shrubs and trees. In contrast, ferns and graminoids were underrepresented in the commercial trade. There was a strong phylogenetic signal associated with commercial availability; some plant families had nearly all or none of their species commercially available. Example families with low representation included Orchidaceae, Potamogetonaceae, Cyperaceae, and fern families. Longer bloomed species were more commercially available, but we did not find differences in availability between early‐ and late‐blooming species. Despite the diversity of the commercial pool in the Midwest, it is an unrepresentative, nonrandom subset of the regional species pool. This finding may promote the mismatch in species diversity and composition between remnant natural habitats and restorations because many specialized species are commercially unavailable to conservation and restoration practitioners. We encourage strategies to promote the availability of underrepresented plant diversity in the commercial species pool.

## INTRODUCTION

Commercial availability of plant species has substantial biodiversity implications. Plant species' commercial availability can facilitate their propagation in residential and urban settings (Zinnen & Matthews, 2022), and drive community composition in urban landscapes (e.g., Cavender‐Bares et al., 2020). Commercial availability also influences the outcomes of conservation projects and ecological restoration. For example, conservation projects, such as reforestation efforts, can be facilitated by the availability of plants in nurseries (Haase & Davis, 2017). Similarly, plant vendors are a source of native plant materials for ecological restoration, particularly in highly impacted landscapes (Zinnen, Broadhurst, et al., 2021). Commercial availability can therefore influence conservation outcomes in both seminatural and human‐dominated contexts.

Despite the critical importance of commercial availability of native plant species for conservation, existing research suggests that most native species are unavailable for purchase. For example, White et al. (2018) studied the species sold by native nurseries throughout the United States, finding that only 26% of all species native to the United States were commercially available, although a substantial 74% of species native to tallgrass prairies were available. Ladouceur et al. (2018) found that only approximately 40% of grassland species were available as seed in Europe. Zinnen and Matthews (2022) found that 44% of species native to the Midwest, USA, were commercially available. While many species are not commercially available at all, even species that can be purchased may have limited availability. For example, Zinnen and Matthews (2022) found that most (~82%) commercially available species were found in fewer than 5% of sampled plant vendors. Consequently, ecological restorations may fail to match the composition or function of reference communities, because many species are difficult to obtain and are therefore not incorporated into restorations (e.g., Barak et al., 2017; Rodrigues et al., 2009).

These and similar studies have described a class of underutilized species, species that are ecologically and functionally valuable to ecosystems, but underrepresented or absent in commercial or restoration settings (Broadhurst et al., 2016; Zinnen, Broadhurst, et al., 2021). Previous studies have indicated that herbaceous species and those with specialized ecological characteristics are less available. In contrast, other species are strongly overrepresented in the commercial trade. For example, overrepresentation of tree species is well documented in neotropical nurseries; *Pinus* species are commercially ubiquitous in Mexico (Ramírez‐Soto et al., 2018), as are pioneer tree species in Atlantic Forests (Rodrigues et al., 2009). In Europe, plant species used as grassland fodder are frequently available, whereas specialist grassland indicator species (i.e., those with high conservation value) are underrepresented (Ladouceur et al., 2018). Although most prairie species can be readily sourced from nurseries in the United States (White et al., 2018), many wetland species cannot (Henry et al., 2024; Kettenring & Tarsa, 2020). There is a near‐complete dearth of some physiognomies in Brazilian native plant nurseries, including herbaceous understory, epiphytic, and liana species (Vidal et al., 2020). Furthermore, plant phenology, specifically flowering time, is a critical aspect of provisioning pollinators with resources (Morellato et al., 2016). However, species available for restoration have been found to be phenologically nonrandom. For example, commercially available prairie seed mixes overrepresent late‐flowering species but underrepresent early blooming species (Havens & Vitt, 2016), perhaps due to early blooming species' low availability or high seed prices (Barak et al., 2022). Furthermore, available species may be longer blooming (Kaul et al., 2023). White et al. (2018) described additional characteristics associated with commercially available species across the United States, including being geographically widespread and having a woody growth form (viz., trees). The lack of commercial availability of some plant species or groups may be explained by several nonmutually exclusive factors. Some species may be more difficult for vendors to source or cultivate, whereas others may lack consumer interest (i.e., demand).

Low commercial representation of specialized or short‐lived plant species might also overlap with phylogenetic lineages. For example, Barak et al. (2017) showed that restored prairies were less diverse than remnants, owing in part to the fact that restoration seed mixes were missing clades found in remnant prairies. Furthermore, sedges (Cyperaceae) are a diverse group of graminoids in North America; despite their potential functional value in wetland and prairie restorations, they are uncommonly included due to inadequate commercial availability (Boeck Crew et al., 2020). Similarly, Ladwig et al. (2020) showed that the composition of savanna seed mixes was phylogenetically clustered compared with remnants. These authors noted that seed mixes lacked several plant families found in remnants (e.g., Ericaceae, pteridophytes), but the mixes overemphasized others (e.g., Poaceae, Lamiaceae).

Despite the importance of commercial availability of native plants for conservation and other goals, few studies have explicitly elucidated the factors associated with commercial availability. This knowledge gap leads to the question: what characteristics are associated with limited commercial availability? Similarly, how representative of the regional flora is the commercial pool? Here, we applied the species pool concept to these questions. The species pool concept describes how a regional native species pool is gradually filtered by ecological factors to form a smaller, local pool of species (Keddy, 1992). Analogously, regional native plant species pools could be filtered by ecological factors that make them difficult or impractical to source or cultivate by vendors, thereby forming a smaller pool of commercially available species.

This study characterized factors associated with representation in the native *commercial species pool* in the Midwestern United States. We investigated species filtration from the regional native pool into the commercial pool by studying species' ecological and evolutionary characteristics (Appendix S1: Figure S1). The commercial species pool is the subset of native plant biodiversity sold by any vendor for a variety of end uses in any material form (Zinnen & Matthews, 2022). The commercial species pool concept is an extension of the restoration species pool, which is the subset of species available seed by vendors for ecological restoration (Kaul et al., 2023; Ladouceur et al., 2018). However, the commercial species pool is more inclusive because it incorporates any material (e.g., seed, plug, potted plant, bare root) and includes plant materials that are not destined for use in ecological restoration (e.g., woody species and specimen plants for native gardens). The commercial species pool is important for understanding native plant availability because different vendor types and material uses can lead to complementary improvements to native plant biodiversity and ecosystem services (Zinnen & Matthews, 2022).

Our overall objective was to identify the ecological characteristics and phylogenetic composition of native species diversity held by commercial vendors. The specific objectives were to (1) identify which ecological factors were associated with commercial presence/absence and frequency; (2) determine whether phylogeny is associated with commercial availability, and, if so, identify clades with high and low availability of their respective species; and (3) compare the phenology of flowering between available and unavailable angiosperms. We hypothesized that four ecological characteristics would be associated with commercial availability: (1) distribution size, (2) growth form, (3) fidelity to wetland habitats, and (4) ecological conservatism, defined as a species' relative intolerance to human impacts and fidelity to natural areas with their historical ecological conditions intact (Swink & Wilhelm, 1979). Similarly, we expected that there would be underrepresented phylogenetic clades that were associated with these characteristics. These four characteristics were selected because they were hypothesized to be associated with the difficulty of sourcing and cultivation by commercial vendors, and because other regional studies (Kaul et al., 2023; Ladouceur et al., 2018; Vidal et al., 2020; White et al., 2018) have suggested that these traits may be relevant to commercial availability. Lastly, based on previous studies, we hypothesized that early and short‐blooming species were less likely to be commercially available.

## METHODS

### Study region

Our study region was seven states in the Midwestern United States: Illinois, Indiana, Iowa, Michigan, Missouri, Minnesota, and Wisconsin. The study area represents a region with intense human impacts (Sanderson et al., 2002), notably, a rapid loss of historical plant communities due to intense agriculturalization in the 19th and 20th centuries (Jackson & Jackson, 2002; Schwartz, 1997). Furthermore, ecological restoration is widely practiced in this region, which has prompted the development of businesses specialized in procuring native plant materials (e.g., Gibson‐Roy, 2018). This context means the Midwest is a particularly strategic location to better understand the factors associated with commercial availability.

### Compiling the native species pool and collecting availability data from plant vendors

To systematically characterize the commercial availability of native plant species, we first had to identify the pool of native species, and then determine which of these species were sold. Therefore, we first compiled a regional species pool for the Midwest by combining state vascular floras from the Universal Floristic Quality Calculator (Freyman et al., 2016) and MNTaxa (Minnesota Department of Natural Resources, 2020)—this list represented a potential maximum for the commercial pool. We included a species in the regional species pool if it was designated as native on at least one state list. Hybrids or species that were not native to any of the seven study states were not included in the analyses. Taxonomy was standardized using the USDA Plants Database (USDA NRCS, 2020; https://plants.sc.egov.usda.gov/). The regional species pool included 2919 species.

Second, we had to determine which of the 2919 species were commercially available. We used a database of 557 plant vendors throughout the Midwest region in the United States (Zinnen & Matthews, 2022) to confirm which species were or were not available. This database was created by screening a listing of nurseries, landscapers, and garden centers from NurseryTrees.com LLC, as well as searching through Plant Information Online (University of Minnesota Plant Information Online, 2020) for plant vendors with online catalogs. This database was composed of data from vendors that sold any living plant material and had detailed species lists (i.e., catalogs or inventories), and a range of vendor types was sampled (e.g., species‐poor local garden centers to diverse native plant nurseries).

Native species sold by these vendors, and when available, the type of material sold to represent each species (e.g., seed, potted, or within a seed mix), were recorded. This database did not include cultivars or species not indigenous to the Midwest region. However, naturally occurring varieties or subspecies that were native to the Midwest were recorded as parental taxa (e.g., *Phlox pilosa* ssp. *fulgida* was recorded as *P. pilosa*). All species, materials sold, and vendor data were collected from February through August 2020.

The database from Zinnen and Matthews (2022) included data on a variety of vendor types with differing target markets, common species, and materials sold. We incorporated data from all vendors and material types into the analysis. This approach avoided difficulties in subjectively determining which vendors or materials should be omitted from analyses. Specifically, we did not want to assume how diverse end users would commercially source their materials. For example, most restoration practitioners probably source most of their materials from speciose native plant nurseries, but they could source trees or native plugs from conventional tree farms or garden centers. To determine commercial availability, we extracted all species that were available as any material and from any type of vendor. Species present in the regional species pool but not present in the database were considered commercially unavailable (i.e., they were not in the commercial species pool).

### Ecological factors associated with commercial pool

For each species in the regional pool, we collected data for four ecological variables, which were hypothesized to associate with two general qualities that could influence commercial availability (Table 1), difficulty of cultivation or sourcing by vendors (supply), and consumer interest (demand).

**TABLE 1:** A summary of the ecological characteristics considered in the study.

Factor	Metric	Values	Hypothesis	Detailed justification
State count	No. (native) inhabited study states	1–7	Larger range size will be positively associated with availability.	A broader range should reflect a species' ability to successfully grow and establish throughout the study region. Conversely, range‐restricted species may not tolerate the climate or soils of many parts of the study region and are likely more difficult to source and cultivate.
Growth habit	Basic physiognomy	Long‐lived forb; long‐lived graminoid; shrub; tree; long‐lived vine; long/short‐lived fern^a^; short‐lived forb; short‐lived graminoid; short‐lived vine	Woody and forb species will be associated with greater availability, whereas graminoids and ferns will be less represented. Short‐lived species of some of the growth forms will be less available.	Other studies of commercially available species (e.g., Hancock et al., 2020; Vidal et al., 2020; White et al., 2018) have shown a consistent bias toward trees, possibly due to their greater ease of cultivation. White et al. (2018) previously showed that ferns and graminoids were underrepresented in the commercial trade, as well as species with short lifespans in the continental United States. Herbaceous short‐lived species may be especially difficult to cultivate by vendors.
Hydrological specialization	Regional wetland indicator status (Reed, 1988)	OBL, FACW, FAC, FACU, UPL	Increasing propensity for wetlands will be negatively associated with availability.	Wetland species are specialized and may pose difficulties to vendors, both in their cultivation and perhaps a more limited market demand. Wetland species are considered underutilized, at least as seed (Kettenring & Tarsa, 2020).
Ecological conservatism	Coefficient of conservatism (Swink & Wilhelm, 1979)	0–10	Increasing ecological conservatism will be negatively associated with availability.	Ecologically conservative species (i.e., those with high *C* values, e.g., >6) may be difficult to cultivate because they are often niche specialists and require historical ecological conditions to thrive (Swink & Wilhelm, 1979). Ecologically conservative species are also regionally rare and limited in distribution (Spyreas, 2019); such species may therefore be obscure to many consumers.
^b^Phenological characteristics	Midpoint blooming date and blooming phenology range	Day of year (1–365)	Later blooming (i.e., those that bloom in the late summer or fall) and longer blooming species will have greater availability than early (spring) and shorter blooming species.	Havens and Vitt (2016) noted that early blooming species were missing in prairie seed mixes. Barak et el. (2022) found that early blooming species were desired by practitioners but insufficiently available. Species that have longer flowering periods might also be more charismatic and given more attention by both commercial vendors and purchasers.

*Note*: These are factors we expected to be associated with commercial availability, as well as specific metrics, hypotheses related to the factors, and a justification for inclusion.

Abbreviations: FAC, facultative; FACU, facultative upland; FACW, facultative wetland; OBL, obligate; UPL, upland.

^a^ Short‐lived ferns or other sporulating plants were combined with long‐lived ferns due to their low sample sizes (*n* = 2).

^b^ Phenological characteristics were only analyzed for angiosperms in the regional pool that had available phenology data from Wilhelm and Rericha (2017).

We expected that greater geographic distributions of native plant species would be positive predictors of both supply and demand, and thus commercial availability. As a coarse proxy of geographic distribution size, for each species in the potential pool, we counted the number of states in the study region (up to seven) to which it was native using the Biota of North America Program (BONAP) county‐level species distribution maps (Kartesz, 2015). BONAP represents the most comprehensive compilation of plant species' records and distributions, and the records are verified by vouchers (Kartesz, 2015). If a species' native status conflicted between data sources (namely, between BONAP and the coefficient of conservatism lists, see below), we also referred to the USDA Plants Database (USDA NRCS, 2020) to establish native status among the study states.

We extracted basic life history and botanical information, namely growth habit and lifespan from the USDA Plants Database and state lists. Lifespans were first recorded as short‐ (annual, biennial, and facultatively annual or biennial species) or long‐lived (strictly perennial) species. Growth habits were recorded as trees, shrubs, vines/lianas, forbs, ferns, and allies (i.e., other vascular sporulating plants), or graminoids (grasses, sedges, and rushes). We then combined lifespan and growth habit to create nine generalized plant types (Table 1). We created these nine plant types because, in our initial analysis, we found strong effects of both lifespan and growth form on availability. However, because growth form and lifespan are inherently nonindependent (e.g., there are no short‐lived trees and shrubs), these variables were combined.

We assessed species' hydrological specialization (i.e., moisture requirements) using wetland status values. Wetland indicator statuses were developed for wetland delineation and reflect an estimated degree of fidelity of plant species to wetland conditions (Reed, 1988). Five categories are used: obligate (OBL), facultative wetland (FACW), facultative (FAC), facultative upland (FACU), and upland (UPL). These data were downloaded from the US Army Corps of Engineers (US Army Corps NWPL, 2020). Because wetland values can vary by region, we preferentially used wetland values of the Midwest region, given its close overlap with the study region. Because some species did not have values since they were on the margins of the Midwest region, some wetland values were used from the Northcentral/Northeast and the Eastern Mountains/Piedmont regions.

We also examined how ecological conservatism might influence commercial availability. Ecological conservatism characterizes a species' fidelity to historical (i.e., remnant) ecological conditions and degree of tolerance to modern anthropogenic disturbances (Spyreas, 2019; Swink & Wilhelm, 1979). Ecological conservatism is also a reflection of a species' perceived conservation value and can be associated with specialized species (Zinnen, Spyreas, et al., 2021). We used coefficients of conservatism (*C* values) to assess whether ecological conservatism was associated with commercial availability. *C* values are expert‐assigned values from 0 to 10, with higher values indicating a more conservative species (Swink & Wilhelm, 1979). *C* values are generally assigned on a state‐by‐state basis. Therefore, we used state *C*‐value lists of seven study states from the Universal Floristic Quality Database (Freyman et al., 2016) and averaged each species' *C* value across the states in which it was found in the regional species pool.

We tested the effect of these ecological characteristics on commercial availability in binary presence (i.e., whether a species was commercially available or not) and frequency (counts, i.e., the number of vendors that sold a particular species) models. We used multivariate logistic regression to predict binary availability, creating multiple generalized linear models (GLMs) of the “binomial” family. We modeled the response variable of commercial presence/absence by creating a global model that included the four predictor variables (plant type, state count, *C* value, and wetland indicator status). We also added a fifth quadratic term in the global model for mean *C* value because exploratory data analysis suggested there was an ostensible hump‐shaped relationship between these variables. We then used dredge() to find which combinations of the predictor variables best explained the data (Bartoń, 2023). Then, once the best model of commercial availability was selected, we identified which variables were the strongest or weakest individual predictors (i.e., univariate models). To model commercial frequency, we created multiple negative binomial models that had different combinations of the four main predictor variables. We used negative binomial models to account for overdispersion (variance is greater than the mean) in the count data. Like the availability analysis, we also included a fifth quadratic term for mean *C* value. For all model comparisons, the best model was selected using the corrected Akaike information criterion (AIC_c_). The best supported models were visualized using ggeffects (Lüdecke, 2018); these plots show predicted probabilities of commercial presence for the logistic regression, whereas frequency predictions were shown as raw counts. We conducted these analyses, as well as the phylogenetic and phenological analyses (see below), in R v. 4.0.2 (R Core Team, 2020).

### Phenological analysis

We also determined whether there was a phenological bias in commercial availability of plant species. Blooming period phenological data were compiled using Wilhelm and Rericha (2017) for 1469 angiosperm species. Because phenology data were available for only about half of the regional species pool, we analyzed phenological bias separately from the four ecological factors. Wilhelm and Rericha (2017) dates were collected in the Chicago region, which represents a rough central location of the study region.

We used binomial linear mixed‐effects models to determine whether commercial availability was associated with two phenological characteristics, midpoint blooming date and blooming phenology range (i.e., the length of time during the growing season when a species could be in flower). We used binomial linear mixed‐effects models to account for the fact that phenology is contingent upon plant type (Appendix S1: Figure S2). Binary availability was our dependent variable, whereas midpoint blooming date and blooming phenology range were fixed effects in two sets of models. We constructed three models: (1) a model with no fixed effect, but with the random effect of plant type (null); (2) a model with random slopes and intercepts; and (3) a model with random intercept only. For all models, the slopes and intercepts were constructed to be correlated. We selected the best mixed‐effects model using AIC_c_.

### Regional phylogenetic tree construction and analysis

We generated a phylogenetic tree of the regional species pool using the R package V.PhyloMaker (Jin & Qian, 2019). V.PhyloMaker is linked to a dated (i.e., known evolutionary distance) mega‐tree compiled for angiosperms and tracheophytes (Smith & Brown, 2018; Zanne et al., 2014), which includes over 74,000 plant species. Tips for a new genus were bound to the half point of the family branch; when the family branch length was longer than two thirds of the whole family branch length, tips of new genera were bound to the upper one third point of the family branch lengths. Species that were not in the list were placed as basal polytomies for their respective genus.

Because the backbone is dependent on taxonomy linked to The Plant List (The Plant List, 2010; http://www.theplantlist.org/), some (*n* = 193) of the species names were converted for this part of the analysis as the initial data set was standardized using the PLANTS Database (USDA NRCS, 2020). We bound 104 species in missing genera to a closely related genus using the genus. bind() function. These were species whose genus was not present in the mega‐tree lists, so their evolutionary distance to other species had to be estimated using the location of a closely related genus. Similarly, we bound 36 species to sister species when they were not present in the mega‐tree since we considered these better estimates than basal polytomies when we had knowledge of their close relationship (e.g., unmatched *Acer nigrum* was considered a variety of *Acer saccharum* in The Plant List, the latter being present in the mega‐tree). Thereafter, a total of 1743 species were linked to the mega‐tree, whereas 1176 species were included in the tree as genus‐ or species‐level polytomies. Consequently, the entire regional tree had 2919 species, which represented a total of 809 genera and 169 families.

To assess the phylogenetic signal of commercial presence, we used the *D* statistic in the R package caper (Orme et al., 2018). The *D* statistic is used for binary traits and assesses whether a trait is phylogenetically random (*D* = 1), nonrandom (*D* < 1), or characteristic of a Brownian motion model (*D* = 0) of trait evolution (Fritz & Purvis, 2010). Several choices can influence the overall structure of a phylogenetic tree, and therefore the resulting phylogenetic findings. For example, the inclusion of nonangiosperms can bias analyses due to their ancient basal status. Furthermore, the 1176 species added as polytomies also changed the tree structure and could have influenced our findings because they were unmatched in the dated mega‐tree. To ensure robustness and generalizability of the results from the total tree, we calculated the phylogenetic signal of commercial availability using three additional trees: a tree consisting of only the species that were found in the V.PhyloMaker mega‐tree, a tree consisting of only angiosperm species, and a genus‐level tree.

To reinforce findings and add practical context to interpreting the regional phylogeny, we also identified which prominent plant families of the Midwest had lower or greater proportions of their species available. In other words, we identified families in the commercial pool that were under‐ or overrepresented. We first considered plant families prominent if they had at least 10 species in the regional species pool, which yielded 63 prominent families. We performed a chi‐square test to determine whether availability was randomly distributed among the families. After the chi‐square test showed significant deviation from null expectations (χ^2^ = 376; *p* < 0.001), we calculated the proportion of species that were commercially available within each prominent family. We then identified the 10 families with the greatest proportions and the 10 families with the lowest proportions.

## RESULTS

Of the 2919 species in the potential native pool of the seven study states, a total of 1277 species (44%) were found in the commercial trade, whereas 1642 species were unavailable. The mean number of occurrences (i.e., the number of vendors selling the species) in the commercial trade per species was 8.82, with a SD of 24.4.

**FIGURE 1:**
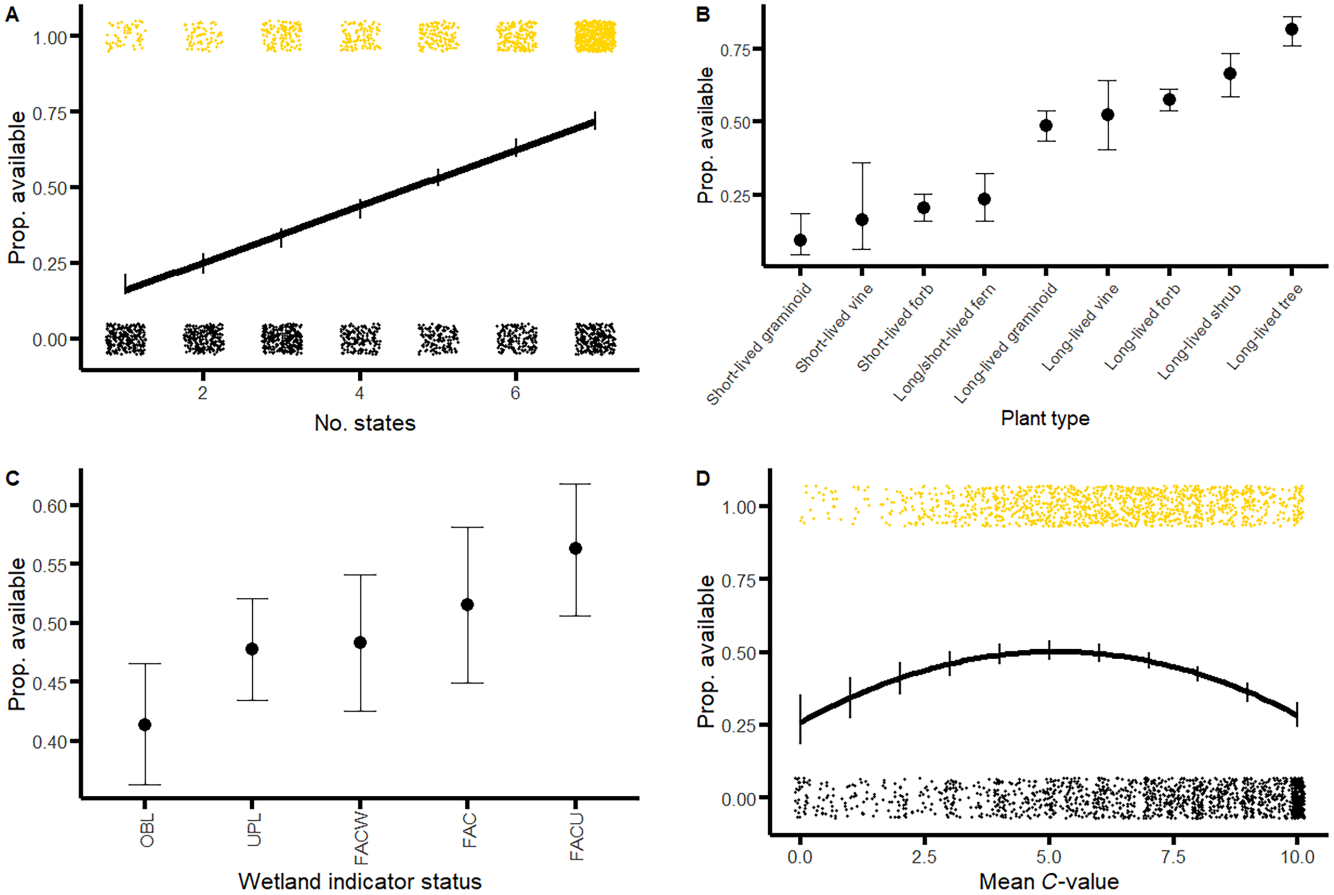
Commercial availability is associated with four ecological factors. Availability for each factor was estimated as proportions from estimated marginal means using ggeffects (Lüdecke, 2018); shown marginal means are from the best supported model that included all four ecological factors, and a quadratic mean *C*‐value term, in a logistic regression. Commercial availability was the highest for species with a greater number of native inhabited states (A), and among long‐lived and woody plant types (B). Availability was also higher for facultative upland species (C), and those with intermediate mean coefficients of conservatism around 3–6 (*C* value; D). For (C), UPL = upland, FACU = facultative upland, FAC = facultative, FACW = facultative wetland, and OBL = obligate wetland. For (A and D), vertical black lines show the 95% CI for the estimated proportions; data points of raw observations of available (1, gold) and unavailable (0, black) species are also shown, with random noise added to points for improved data visualization. Dots in (B and C) represent the marginal mean proportion of availability, and lines indicate 95% CIs.

For commercial availability (presence/absence), the best supported model included all four ecological characteristics and a quadratic mean *C*‐value term (AIC_c_ = 3076; weight = 0.906; Table 2, Figure 1). The second best model had some support (AIC_c_ = 3081; weight = 0.088), which included plant type, state count, and mean *C*‐value terms, but not wetland indicator status. All the other models that included at least one predictor outperformed the null model (ΔAIC_c_ > 39). When only single variables were used to explain presence/absence, state count was the strongest predictor (AIC_c_ = 3468.4; weight >0.999), followed by plant type (AIC_c_ = 3603.7; ΔAIC_c_ = 135). The weakest single predictor was wetland indicator status (AIC_c_ = 3867.2), although it still markedly outperformed the null model (ΔAIC_c_ = 39).

**TABLE 2:** Comparisons of models that explain commercial availability (presence/absence) and frequency.

	Model	df	AIC_c_	ΔAIC_c_	Weight
Availability	A ~ S + P + W + C + C^2^	16	3039.1	0.0	0.991
	A ~ S + P + C + C^2^	12	3048.6	9.5	0.009
	A ~ S + P + W + C^2^	15	3065.2	26.1	<0.001
	A ~ S + P + C^2^	11	3072.4	33.3	<0.001
	A ~ S + P + W + C …	15	3075.8	36.7	<0.001
	A ~ 1 (null)	1	3906.5	867.4	<0.001
Frequency	F ~ S + P + W + C + C^2^	17	12,470.9	0.0	>0.999
	F ~ S + P + W + C^2^	16	12,494.2	23.3	<0.001
	F ~ S + P + W + C	13	12,517.3	46.4	<0.001
	F ~ S + P + C^2^	12	12,526.3	55.4	<0.001
	F ~ S + P + C …	12	12,538.5	67.5	<0.001
	F ~ 1 (null)	2	13,254.1	783.2	<0.001

*Note*: Availability models were binomial GLMs; frequency models were negative binomial models. For brevity, only the five best models including ecological factors are shown, and the null model as a sixth model for comparison. For these analyses, 79 species were excluded because they did not have *C* values; included are 2840 species from the total species pool. Model terms: A = availability; F = frequency; S = state count; P = plant type; W = wetland indicator status; *C* = mean *C* value. See Appendix S1: Table S1 for all models.

Abbreviations: AIC_c_, corrected Akaike information criterion; GLM, generalized linear model.

Availability strongly increased with the number of states in which a species was native (Figure 1A). Species only native to a single state were predicted to have less than a 20% chance of being commercially available, whereas species found in all seven states in the Midwest had nearly an 80% chance. There was also a strong effect of plant type. Availability was generally much lower for short‐lived plant types (Figure 1B), and this effect was most pronounced in short‐lived graminoids and vines. Ferns and other sporulating plants also had low availability that was on parity with short‐lived plant types. Trees were the most likely to be available (nearly 80%), followed by shrubs (~60%), while long‐lived vines, forbs, and graminoids had intermediate levels of availability. The two expert‐based ecological indicators, wetland indicator status and mean *C* value, were also associated with availability, although model selection suggested these variables were less important than state count or plant type (Figure 1C,D). Obligate wetland species were the least likely to be available (~35%), whereas FACU species were the most likely to be available (~50%). Intermediately conservative species were the most available, whereas availability sharply declined at the lowest and highest mean *C* values, meaning species least and most sensitive to human impacts were the least available.

For commercial frequency across analyses, our findings generally matched those we found regarding commercial availability. In other words, the most commercially available groups of species were also the most frequent. The best supported model included all four ecological characteristics and a quadratic mean *C*‐value term (Table 2). All models that included at least one ecological characteristic outperformed the null model (Table 2; Appendix S1: Table S1): the single best predictor was state count, and the single worst predictor was wetland indicator status (Appendix S1: Table S1). Frequency was positively associated with state count (Appendix S1: Figure S3). Of the plant types, some (e.g., short‐lived graminoids, short‐lived forbs, and ferns) were predicted to have frequencies near zero. Trees and shrubs were the most frequent, expected to be found in more than 30 and 10 vendors, respectively. Facultatively upland species were encountered the most often, whereas obligate wetland species were the least frequent. Like availability, the prediction of frequency by mean *C* value was hump‐shaped, meaning species with intermediate *C* values were the most frequently sold.

**FIGURE 2:**
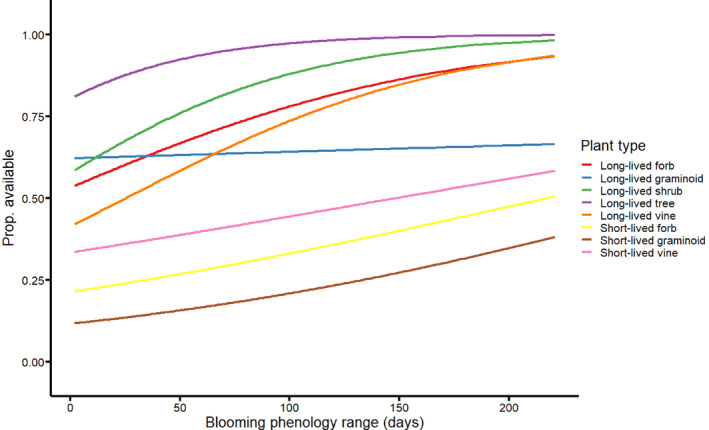
Greater availability is associated with longer blooming phenology ranges. Curves represent predicted availability of a species based on a binomial linear mixed‐effects model that incorporated blooming phenology range when controlling for plant type. Plant type was included in the model as a random effect because of the substantial phenological differences between short‐ and long‐lived species, and among different growth forms (Appendix S1: Figure S2). Proportions are from estimated marginal means using ggeffects (Lüdecke, 2018).

For blooming phenology range, the optimal mixed‐effects structure included a random slope and intercept for blooming phenology range to predict availability (Appendix S1: Table S2; AIC_c_ = 1775; weight = 0.78). Blooming phenology range had a modest yet positive influence on availability. Furthermore, this optimal model that included blooming phenology range substantially outperformed the models where blooming phenology range was included with a random intercept only (ΔAIC_c_ = 2.5) and the null model (ΔAIC_c_ = 16). In other words, when controlling for plant type, longer blooming species had greater estimated probabilities of being commercially available. However, the strength and direction of the relationship varied by plant type (Figure 2). The longest blooming woody (shrub and tree), long‐lived forb, and long‐lived vine species had high estimates of availability, exceeding proportions of 0.80 with >150 blooming days, although these effects saturated. One noteworthy finding was that blooming phenology range had little effect on availability for long‐lived graminoids. For blooming midpoint, the optimal mixed‐effects structure included a random slope and intercept for blooming midpoint to predict availability (Appendix S1: Table S2; AIC_c_ = 1789; weight = 0.55). However, neither this model nor the second best model that included blooming midpoint and a random intercept significantly outperformed the null model (ΔAIC_c_ < 2).

**FIGURE 3:**
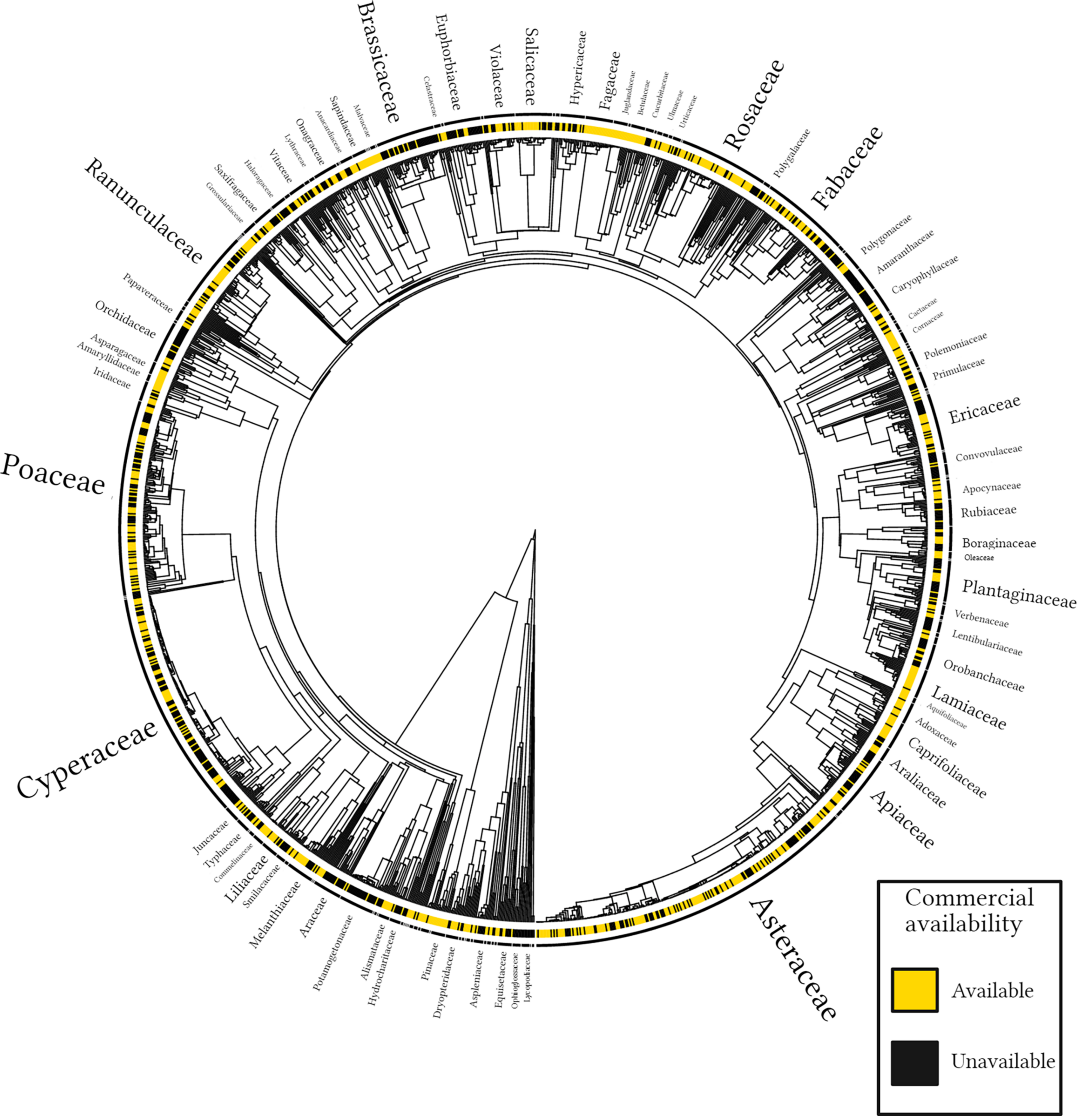
A phylogeny of commercial availability of each species (tree tip) in the Midwestern United States. Colors at the tips indicate whether a species is available or unavailable in the commercial species pool. Black bars outside color‐coded availability represent a plant family clade. For readability, this phylogeny only included the species that were present in the V.PhyloMaker dated mega‐tree (*n* = 1743 species), and some family names with few species are omitted. This figure was created using the R package diversitree (FitzJohn, 2012) and BioRender. Font size is approximately scaled for the number of species in the clade.

There was a significant phylogenetic signal of species that were commercially present or absent (*D* = 0.66; *p* < 0.001 relative to random model). This finding was robust across the analyses using the angiosperm tree (*D* = 0.68; *p* < 0.001 relative to random model), the subset of the tree that was matched and dated (Figure 3; *D* = 0.66; *p* < 0.001 relative to random), and the genus‐level tree (*D* = 0.75; *p* < 0.001 relative to random). Table 3 summarizes the prominent families that had higher and lower commercial representation than average. Notable families with sporadic or low commercial representation included Orchidaceae, Potamogetonaceae, Brassicaceae, Euphorbiaceae, Cyperaceae, Rubiaceae, Araceae, and extreme basal families, such as Isoetaceae (Table 3, Figure 3). We also noticed that partially or fully parasitic genera (e.g., *Cuscuta*, *Orobanche*) within some families (e.g., Convolvulaceae, Orobanchaceae) had low commercial representation. In fact, in follow‐up analysis, we found that hemi‐ and holoparasitic species (Appendix S1: Table S3) were significantly less likely to be available than expected when availability across the total data set was considered (exact binomial test one‐tailed: *p* = 0.004). Well‐represented families among the commercial species pool included Fagaceae, Pinaceae, Lamiaceae, Juglandaceae, and Betulaceae (Table 3, Figure 3). The species‐rich *Rubus* and *Crataegus* genera make Rosaceae relatively incomplete with respect to commercial availability (data not shown), despite the family's well‐represented status in the matched tree (Figure 3).

**TABLE 3:** A summary of the greatest and least represented prominent plant families in the Midwest based on commercial availability.

Family	Expected richness	Richness available	Family richness	Proportion available
Highly available plant families				
Cornaceae	4	10	10	1.00
Pinaceae	4	10	10	1.00
Fagaceae	11	25	26	0.96
Betulaceae	6	12	13	0.92
Juglandaceae	5	11	12	0.92
Sapindaceae	5	10	12	0.83
Adoxaceae	6	12	15	0.80
Malvaceae	6	11	15	0.73
Asparagaceae	5	8	11	0.73
Caprifoliaceae	10	16	23	0.70
Highly unavailable plant families				
Euphorbiaceae	14	4	33	0.12
Brassicaceae	23	6	53	0.11
Potamogetonaceae	12	3	29	0.10
Aspleniaceae	4	1	10	0.10
Solanaceae	6	1	15	0.07
Convolvulaceae	9	1	20	0.05
Amaranthaceae	10	1	24	0.04
Lentibulariaceae	5	0	11	0.00
Lycopodiaceae	8	0	18	0.00
Ophioglossaceae	11	0	25	0.00

*Note*: The expected richness column is the number of species expected to be available, assuming that the number of available species across all prominent families was proportionally equivalent; richness available is the number of species in the family that were commercially available.

## DISCUSSION

### Overview

The commercial species pool in the Midwestern United States is missing more than half of the regional flora. Availability is not random; some phylogenetic clades' ecological factors were associated with lesser or greater commercial presence and frequency. Generally, the species excluded (i.e., filtered) from the commercial pool could be categorized into two nonmutually exclusive groups: niche‐specialized (e.g., conservative, or obligate wetland or upland species) and ruderal (colloquially described as “weedy”) species. The former may be more challenging to cultivate and source, whereas the latter are less likely to generate consistent consumer interest (see *Caveats* below). In contrast, there was evidence based on both the ecological factors and phylogenetic analyses that woody species are well‐represented in the commercial trade.

### “Winners” and “losers” of the commercial species pool

These results suggest that vendors in the Midwestern United States hold many species characteristic of the region, specifically common trees, and grassland and woodland wildflowers. Such species are valuable in residential or restoration contexts, whether for aesthetic or biodiversity‐intended end uses. Nevertheless, there are clear groups of “winner” and “loser” plants in the commercial species pool. Our broad findings were remarkably congruent with those of Kaul et al. (2023), who reported that intermediately conservative, wide‐ranged, and showy species had the greatest availability in native seed mixes of the Midwest.

We identified four ecological factors that were strong associates of commercial availability. Distribution size as estimated by state presence was the strongest individual predictor of both availability and frequency, such that species with smaller state counts were the least available. Species restricted to single or few states may not be easily cultivated or sourced by vendors, even within their state of origin. Geographic limitation may fate these species to obscurity or logistical difficulties in their procurement. Plant type was the second strongest predictor, and many herbaceous physiognomies, especially for sporulating and short‐lived species, were underrepresented in the commercial pool. Other studies have also documented low availability or utilization of short‐lived species (de Queiroz et al., 2021; Kaul et al., 2023; Lesage et al., 2018), perhaps because of more difficult management with their temporary lifespan. In contrast, like other studies (Vidal et al., 2020; White et al., 2018), woody species were well‐represented, with trees and shrubs being especially frequent in our data set (Appendix S1: Figure S3). Third, the species most specialized to wetlands were the least available. This is consistent with the idea that wetland species availability and sourcing may lag behind other plant community types (Kettenring & Tarsa, 2020). Similarly, in the Intermountain West, Henry et al. (2024) showed that specialized wetland species (e.g., aquatic or emergent species) were rarely sold by plant vendors compared with terrestrial species. Lastly, the least (*C* value <4) and most (*C* value >6) ecologically conservative species were generally excluded from the commercial species pool. Conservative species have been shown to have more specific niche requirements and limited distributions (Spyreas, 2019; Zinnen, Spyreas, et al., 2021)—characteristics that may make these species difficult to source, cultivate, and generate market (i.e., purchasers) interest. Furthermore, less conservative species may overlap with “weedier” characteristics like short lifespan, fast growth, and greater dispersal capability (Ficken & Rooney, 2020). Thus, the least conservative “weedy” species may be impractical to cultivate (e.g., ruderal species) and are likely avoided as targets for ecological restoration or horticultural plantings.

There were also phenological signals to commercial availability. For many plant species (viz., forbs), flowering phenology is important for pollinator conservation because of its influence on resource availability (Morellato et al., 2016). Longer blooming species were more likely to be available (Figure 2). Kaul et al. (2023) also showed that longer blooming species in the Midwest were more likely to be available and found in more vendors, which they interpreted as support for the “showiness” hypothesis of commercial availability. Similarly, we think this finding was driven by two causes. First, consistent with the “showiness” interpretation, longer blooming species will probably be considered more attractive to both plant vendors and their customers. Second, there may be a sampling bias toward well‐known, common, and charismatic plant species that are likely to be more commercially available in our phenology data source (Wilhelm & Rericha, 2017). We did not find evidence of an availability bias based on blooming midpoint. Nevertheless, just because early‐ and late‐blooming species had similar levels of availability, this does not necessarily mean that utilization of native species will be phenologically unbiased (e.g., Havens & Vitt, 2016; see *Caveats*).

Unsurprisingly, we also found strong evidence that commercial presence was phylogenetically nonrandom. To our knowledge, this represents the first comprehensive phylogenetic characterization of a commercial species pool. Phylogeny reflects species' evolutionary histories and can be a useful proxy and predictor of functional and ecological processes (Srivastava et al., 2012). Because the ecological characteristics in this study are themselves interrelated and constrained by phylogeny, less available clades tended to consist of both specialized and short‐lived species. Some clades highlighted in Figure 3 and Table 3 suggest there are other unmeasured but potentially influential factors that affect availability. For example, propagule size is likely a barrier for cultivating many species, and unsurprisingly, sporulating families (e.g., ferns) or those with dust‐like seeds (e.g., orchids) were less represented. Similarly, “showiness” or “charisma” are multifaceted and not straightforward to measure but are likely important latent factors to explain some of the best represented clades (Kaul et al., 2023). It is notable that well‐represented herbaceous families Asteraceae and Lamiaceae are colorful and have large blooms, whereas woody families like Fagaceae or Juglandaceae are attractive shade trees when mature. Collectively, our findings illustrate that the commercial pool is not only ecologically and taxonomically incomplete (Zinnen & Matthews, 2022), but it also does not reflect coinciding, broader levels of biodiversity.

### Implications for conservation and native plant utilization

Most species native to the Midwest were not available in any capacity. Some of these large plant groups that are excluded from the commercial pool could be useful in native landscaping or restoration (Box 1). This begs the question: what consequences arise from the commercial unavailability of this biodiversity? This question is perhaps most relevant to restoration practitioners. Commercial sourcing of plant materials is especially critical to ecological restoration when landscape factors limit the natural dispersal of desirable species, or where practitioners are unable to source plant materials from natural habitats (Zinnen, Broadhurst, et al., 2021). Low representation of plant biodiversity in the commercial trade can, therefore, fundamentally limit the composition of restorations. In other words, it will generally be impossible for restoration practitioners to recreate reference plant communities if vendors are their only sources of plant materials. And, in turn, restorations may overemphasize biodiversity that is readily available or perhaps more practical to both producers and customers (e.g., Luong et al., 2023). Because practitioners commonly source materials from vendors in the Midwest, other studies have documented that important components of natural areas are absent from Midwestern restorations, such as hemiparasitic species in prairie restorations (Barak et al., 2017, 2022) or shade‐tolerant species in compensatory wetland restorations (Tillman & Matthews, 2023). Studies from Latin America (Ramírez‐Soto et al., 2018; Vidal et al., 2020) and others from North America (Kaul et al., 2023; White et al., 2018) have also highlighted similar commercial favoritism and absence of large portions of the regional flora. These studies highlight the possibility for homogenization of restoration biodiversity or outcomes, in part due to differences in commercial availability (Holl et al., 2022).

BOX 1 **Specific cases and discussion of underutilized species**

**FIGURE 4:**
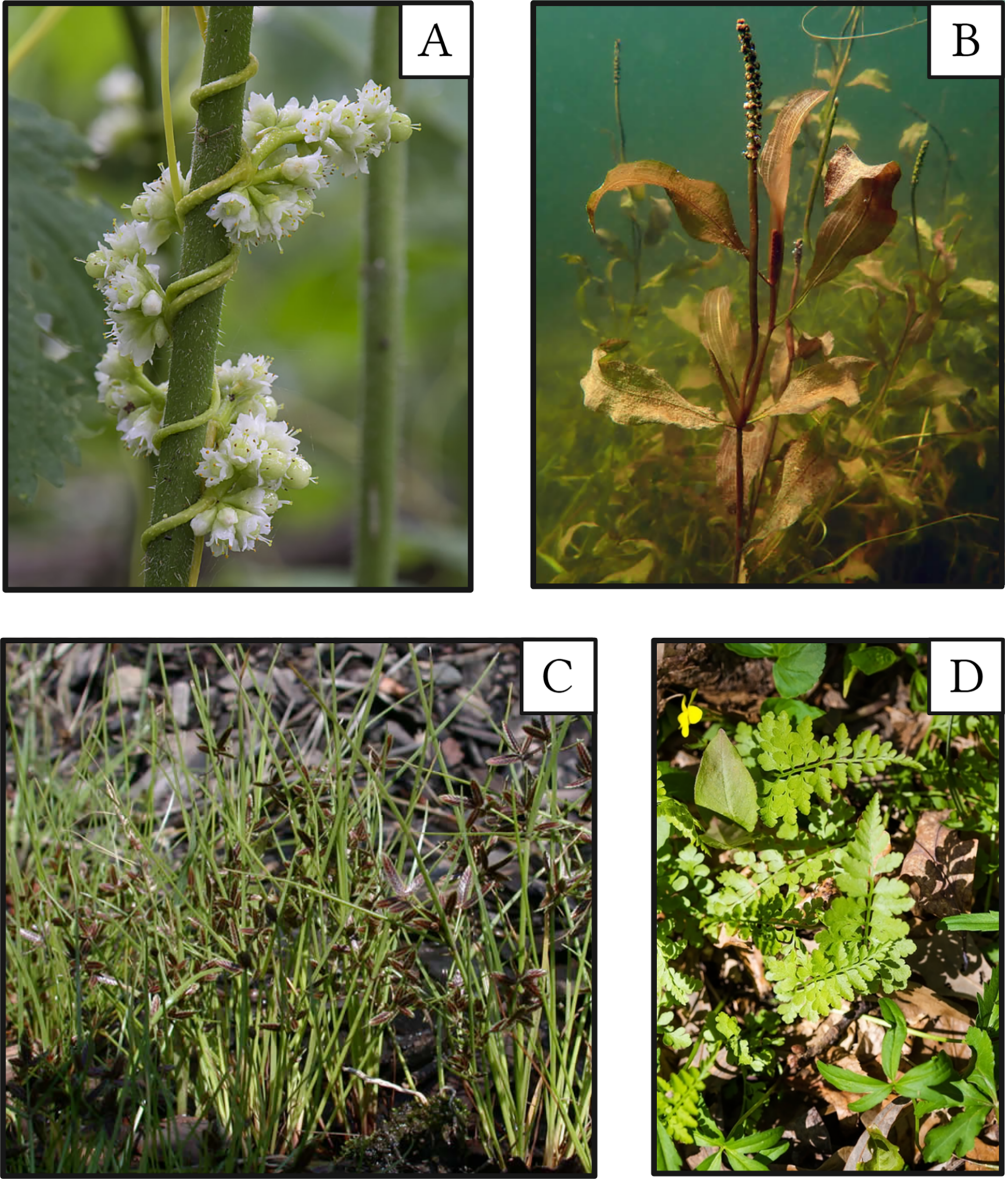
Specific cases where unavailability in the commercial trade can prevent the biodiversity benefits of valuable species and genera from being realized, with specific emphases on ecological restoration or native plant gardening. Pictured are *Cuscuta gronovii* (A), *Potamogeton illinoensis* (B), *Cyperus bipartitus* (C), and *Woodsia obtusa* (D). Photo credits: John Thayer (A) and Peter Dziuk (B and C) with minnesotawildflowers.info, and Melissa McMasters (D).

This study highlights numerous specific examples of species that are potentially suitable for inclusion in the commercial species pool. Numerous poorly available or unavailable groups of species could be underutilized components of native landscaping or ecological restoration (Figure 4) and feasible for cultivation.

Species in the genus *Cuscuta* are members of the underrepresented Convolvulaceae family (Table 3, Figure 4A). They are short‐lived vines that are obligate parasites to aboveground host tissue. Despite their purported risks as agricultural pests, parasitic plants such as *Cuscuta* could have excellent value in conservation and restoration because they can promote coexistence, increase interspecific interactions and connectivity, and provide direct and indirect resources to animals (Těšitel et al., 2021). The family Potamogetonaceae has extremely low commercial representation. Species in *Potamogeton* and related genera are conservative, obligate wetland specialists, and potentially important contributors to wetland function and structure (Figure 4B). Perhaps these species can have restricted but realistic niche end uses, such as some wetland (e.g., lakeshore) restorations, or for use in ornamental ponds in lieu of non‐native plant taxa. *Cyperus* species, some of which are short‐lived, are typically wetland specialists in Cyperaceae inhabiting shorelines and seasonally wet areas (Figure 4C). Their often‐diminutive form is easy to overlook, but they may be candidates for some wetland seed mixes. Although commercially rare, short‐lived species like *C. bipartitus* can provide valuable and timely resources (seed and foliage) as food for waterfowl, and they are capable of temporarily occupying gaps from disturbance. Ferns and sporulating plants are highly underrepresented in the commercial pool. Many sporulating species are undoubtedly difficult to cultivate due to their slow growth, specialized growth conditions, and complex life cycle (e.g., gametophyte phase, especially those that require mycorrhizal fungi). However, some unavailable ferns like *Cystopteris protrusa* (Figure 4D) have a relatively widespread range and broad habitat conditions and may be feasible to cultivate via rhizomes. This species and other ferns could contribute to understory structure in woodland restorations or be attractive accent plants in native shade gardens. However, some ferns may require more advanced cultivation techniques (e.g., tissue culture) for vendors to consider them commercially viable.

Low commercial representation of certain groups of species fundamentally limits options for restoration and native landscaping. Restoration policies advocating for greater functional and lifeform diversity, as well as consumer awareness of the value of these overlooked species, could stoke future demand. Collaborations between habitat managers and plant vendors could provide opportunities to source commercially underrepresented plant biodiversity (Nevill et al., 2016; Zinnen, Broadhurst, et al., 2021).

In addition to constraining options for ecological restoration, commercially unavailable plant biodiversity may also negatively impact native landscaping and gardening. Because our data were primarily sourced (~70%) from conventional plant vendors (e.g., general nurseries and tree farms, Zinnen & Matthews, 2022), our results about frequency reveal how the commercial pool influences native gardening and landscaping in the Midwest. Woody species were by far the most prevalent across all plant vendors. Thus, we suspect that widespread woody plants (namely, dependable native shade trees) are the only consistently well‐utilized group of noncultivar native plants for gardening and native landscaping. The lack of commercially available native species may encourage gardeners to purchase alternatives with less favorable biodiversity values, such as non‐native species or cultivars of native species. Non‐native and native cultivar plants can provide resources for wildlife or pollinators, but their benefits to fauna (e.g., pollinators) can be less than wild‐type native plants (Tallamy et al., 2021; White, 2016), and these plants can pose a risk of becoming invasive (Van Kleunen et al., 2018). There are benefits to improving the availability of some commercially unavailable or rare species. Specifically, native species have vast aesthetic variation, giving many species unexplored potential as garden and landscaping options, and they may be more sustainable to produce than traditional ornamentals (Darras, 2020; Box 1).

Despite these concerns about the limitations of the commercial species pool, it is unrealistic to expect that all species in a region should be commercially available. Many species are unlikely to generate demand due to being considered weedy or unattractive, and many would clearly be too inconvenient to source and cultivate due to their ecologies (Zinnen & Matthews, 2022). Furthermore, some species‐rich groups (e.g., *Carex*, *Crataegus*, and *Rubus*) are composed of species with low ecological and functional distinctiveness; in such cases, having a wide representation of these species may be unimportant when the commercial trade hosts a few quintessential examples (see Violle et al., 2017). While the commercial pool in the Midwest cannot be called representative, it is highly diverse, and the plant vendors forming it have a more robust industry compared with other regions (cf. Gibson‐Roy, 2018; Hancock et al., 2020; Vidal et al., 2020). Furthermore, these vendors provide plant materials for enhancing plant biodiversity across the region (Gibson‐Roy, 2018; Zinnen, Broadhurst, et al., 2021). Future research could identify which native species have unrealized potential for use in ecological restoration or general landscaping.

### Caveats

There are some limitations to this study and more research on the topic is needed. First, our assessment of representation within the commercial pool was limited to binary availability and frequency among plant vendors. Although these variables are informative, they still do not capture fine‐scale information about the gradient of commercial availability. For example, *Gentiana puberulenta* is available in the commercial trade, but only in small volumes, as seed packets from a select few nurseries. Thus, while the species is technically represented in the commercial pool, it is effectively unavailable at appreciable amounts for the purposes of widespread home gardening or ecological restoration; likewise, its commercial availability would only have a marginal influence on its presence in the landscape. As another example, Havens and Vitt (2016) documented that early (spring) blooming species were underrepresented in seed mixes. In our results, when we corrected for plant type, we did not find a significant effect of blooming time on availability. This discrepancy can be understood by contrasting the grain of the two analyses: Havens and Vitt (2016) examined the composition of specific seed mixes for a specific end use (prairie restoration), whereas this study was conducted across a regional flora, across any material types, which made our characterizations of commercial availability more lenient.

Furthermore, our study examined several levels of biodiversity (species, ecological, and phylogenetic), but additional components of biodiversity could be explored in future studies. Specifically, intraspecific genetic diversity, although beyond the scope of this study, is important for ensuring the success and integration of plants in wild settings (Nevill et al., 2016). Because most available species are infrequent among plant vendors (Zinnen & Matthews, 2022; Appendix S1: Figure S3), we suspect that obtaining local ecotypes and capturing the genetic diversity found in the wild would be impractical or impossible for most available species. We concur with White et al. (2018) and suggest that data are needed to screen the genetic diversity and representativeness of the commercial species pool compared with populations in wild settings.

## CONCLUSIONS

Our results provide a comprehensive overview of what is commercially available in the Midwest. The phylogenetic, ecological, and phenological composition of the commercial species pool is nonrandom and inequitable. Specialized and short‐lived (often “weedy”) species and clades were filtered from the commercial pool, whereas families containing woody species and showy forbs were overrepresented. Supply and demand are proximate causes of these patterns, as both factors will influence the biodiversity within the commercial pool. Yet, our hypotheses and results cannot directly tease apart the relative importance of supply or demand on commercial availability, and doing so is not straightforward. This is because supply among vendors—and their capability to feasibly provide materials—is fundamentally entangled with demand on the consumer side. For example, some species (e.g., a short‐lived parasitic species that is native to a section of a single state) would undoubtedly present a challenge to vendors for sourcing and cultivation, and such species would simultaneously correspond to less consumer interest. This presents an opportunity in future studies, however, to clarify the underlying reasons for why we found such strong associates of commercial availability.

## AUTHOR CONTRIBUTIONS

Jack Zinnen created both the commercial and regional species pool databases, compiled the ecological, phenological, and phylogenetic data, analyzed the data, and drafted the manuscript. Rebecca S. Barak and Jeffrey W. Matthews helped design the study. All authors assisted in editing the manuscript.

## CONFLICT OF INTEREST STATEMENT

The authors declare no conflicts of interest.

## Supporting information


Appendix S1:

## Data Availability

Data (Zinnen et al., 2024) are available in the Illinois Data Bank at https://doi.org/10.13012/B2IDB-1143125_V1.

## References

[eap3070-bib-0001] Barak, R. S. , Z. Ma , L. A. Brudvig , and K. Havens . 2022. “Factors Influencing Seed Mix Design for Prairie Restoration.” Restoration Ecology 30: e13581. 10.1111/rec.13581.

[eap3070-bib-0002] Barak, R. S. , E. W. Williams , A. L. Hipp , M. L. Bowles , G. M. Carr , R. Sherman , and D. J. Larkin . 2017. “Restored Tallgrass Prairies Have Reduced Phylogenetic Diversity Compared with Remnants.” Journal of Applied Ecology 54: 1080–1090. 10.1111/1365-2664.12881.

[eap3070-bib-0003] Bartoń, K. 2023. “MuMIn: Multi‐Model Inference.” R Package Version 1.47.5. https://cran.r-project.org/web/packages/MuMIn/index.html

[eap3070-bib-0004] Boeck Crew, C. M. , M. C. Myers , M. E. Sherrard , K. J. Elgersma , G. A. Houseal , and D. D. Smith . 2020. “Stratification and Perigynia Removal Improve Total Germination and Germination Speed in 3 Upland Prairie Sedge Species.” Native Plants Journal 21: 120–131. 10.3368/npj.21.2.120.

[eap3070-bib-0005] Broadhurst, L. M. , T. A. Jones , F. S. Smith , T. North , and L. Guja . 2016. “Maximizing Seed Resources for Restoration in an Uncertain Future.” BioScience 66: 73–79. 10.1093/biosci/biv155.

[eap3070-bib-0006] Cavender‐Bares, J. , J. Padulles Cubino , W. D. Pearse , S. E. Hobbie , A. J. Lange , S. Knapp , and K. C. Nelson . 2020. “Horticultural Availability and Homeowner Preferences Drive Plant Diversity and Composition in Urban Yards.” Ecological Applications 30: 1–16. 10.1002/eap.2082. 31971651

[eap3070-bib-0008] Darras, D. I. 2020. “Implementation of Sustainable Practices to Ornamental Plant Cultivation Worldwide: A Critical Review.” Agronomy 10: 1570. 10.3390/agronomy10101570.

[eap3070-bib-0009] de Queiroz, T. , S. Swim , P. L. Turner , and E. A. Leger . 2021. “Creating a Great Basin Native Annual Forb Seed Increase Program: Lessons Learned.” Native Plants Journal 22: 90–102. 10.3368/npj.22.1.90.

[eap3070-bib-0010] Ficken, C. D. , and R. C. Rooney . 2020. “Linking Plant Conservatism Scores to Plant Functional Traits.” Ecological Indicators 115: 106376. 10.1016/j.ecolind.2020.106376.

[eap3070-bib-0011] FitzJohn, R. G. 2012. “Diversitree: Comparative Phylogenetic Analyses of Diversification in R.” Methods in Ecology and Evolution 3: 1084–1092. 10.1111/j.2041-210X.2012.00234.x.

[eap3070-bib-0012] Freyman, W. A. , L. A. Masters , and S. Packard . 2016. “The Universal Floristic Quality Assessment (FQA) Calculator: An Online Tool for Ecological Assessment and Monitoring.” Methods in Ecology and Evolution 7: 380–383. 10.1111/2041-210X.12491.

[eap3070-bib-0065] Fritz, S. A., and A. Purvis . 2010. “Selectivity in Mammalian Extinction Risk and Threat Types: A New Measure of Phylogenetic Signal Strength in Binary Traits.” Conservation Biology 24: 1042–1051. 10.1111/j.1523-1739.2010.01455.x. 20184650

[eap3070-bib-0013] Gibson‐Roy, P. 2018. “Restoring Grassy Ecosystems–Feasible or Fiction? An Inquisitive Australian's Experience in the USA.” Ecological Management and Restoration 19: 11–25. 10.1111/emr.12327.

[eap3070-bib-0015] Haase, D. L. , and A. S. Davis . 2017. “Developing and Supporting Quality Nursery Facilities and Staff Are Necessary to Meet Global Forest and Landscape Restoration Needs.” Reforesta 4: 69–93. 10.21750/REFOR.4.06.45.

[eap3070-bib-0016] Hancock, N. , P. Gibson‐Roy , M. Driver , and L. M. Broadhurst . 2020. The Australian Native Seed Sector Survey Report. Canberra: Australian Network for Plant Conservation.

[eap3070-bib-0017] Havens, K. , and P. Vitt . 2016. “The Importance of Phenological Diversity in Seed Mixes for Pollinator Restoration.” Natural Areas Journal 36: 531–537. 10.3375/043.036.0418.

[eap3070-bib-0018] Henry, A. L. , R. Robinson , K. Sinnott , M. Brunson , A. Ernst , E. Tarsa , and K. M. Kettenring . 2024. “Got Plants? Availability of and Challenges to Production of Native Plants for Wetland Restoration.” Restoration Ecology 32: e14120. 10.1111/rec.14120.

[eap3070-bib-0020] Holl, K. D. , J. C. Luong , and P. H. S. Brancalion . 2022. “Overcoming Biotic Homogenization in Ecological Restoration.” Trends in Ecology & Evolution 37: 777–788. 10.1016/j.tree.2022.05.002. 35660115

[eap3070-bib-0021] Jackson, D. L. , and L. L. Jackson . 2002. The Farm as Natural Habitat: Reconnecting Food Systems with Ecosystems. Washington DC: Island Press.

[eap3070-bib-0022] Jin, Y. , and H. Qian . 2019. “ *V.PhyloMaker*: An R Package that Can Generate Very Large Phylogenies for Vascular Plants.” Ecography 42: 1353–1359. 10.1111/ecog.04434.

[eap3070-bib-0023] Kartesz, J. T. 2015. The Biota of North America Program (BONAP). Chapel Hill, NC: North American Plant Atlas. http://www.bonap.net/tdc.

[eap3070-bib-0024] Kaul, A. D. , M. Barash , and M. A. Albrecht . 2023. “Common, Showy, and Perennial Species Dominate a Restoration Species Pool.” Restoration Ecology 31: e13969. 10.1111/rec.13969.

[eap3070-bib-0025] Keddy, P. A. 1992. “Assembly and Response Rules: Two Goals for Predictive Community Ecology.” Journal of Vegetation Science 3: 157–164. 10.2307/3235676.

[eap3070-bib-0026] Kettenring, K. M. , and E. E. Tarsa . 2020. “Need to Seed? Ecological, Genetic, and Evolutionary Keys to Seed‐Based Wetland Restoration.” Frontiers in Environmental Science 8: 109. 10.3389/fenvs.2020.00109.

[eap3070-bib-0027] Ladouceur, E. , B. Jiménez‐Alfaro , M. Marin , M. De Vitis , H. Abbandonato , P. P. M. Iannetta , C. Bonomi , and H. W. Pritchard . 2018. “Native Seed Supply and the Restoration Species Pool.” Conservation Letters 11: e12381. 10.1111/conl.12381. 29937920 PMC5993272

[eap3070-bib-0028] Ladwig, L. M. , C. R. Zirbel , Q. M. Sorenson , and E. I. Damschen . 2020. “A Taxonomic, Phylogenetic, and Functional Comparison of Restoration Seed Mixes and Historical Plant Communities in Midwestern Oak Savannas.” Forest Ecology and Management 466: 118122. 10.1016/j.foreco.2020.118122.

[eap3070-bib-0029] Lesage, J. C. , E. A. Howard , and K. D. Holl . 2018. “Homogenizing Biodiversity in Restoration: The “Perennialization” of California Prairies.” Restoration Ecology 26: 1061–1065. 10.1111/rec.12887.

[eap3070-bib-0030] Lüdecke, D. 2018. “Ggeffects: Tidy Data Frames of Marginal Effects from Regression Models.” Journal of Open Source Software 3: 772.

[eap3070-bib-0031] Luong, J. C. , D. M. Press , and K. D. Holl . 2023. “Lessons Learned from an Interdisciplinary Evaluation of Long‐Term Restoration Outcomes on 37 Restored Coastal Grasslands in California.” Biological Conservation 280: 109956. 10.1016/j.biocon.2023.109956.

[eap3070-bib-0032] Minnesota Department of Natural Resources . 2020. “MNTaxa: The State of Minnesota Vascular Plant Checklist.” https://www.dnr.state.mn.us/eco/mbs/plant-lists.html.

[eap3070-bib-0033] Morellato, L. P. C. , B. Alberton , S. T. Alvarado , B. Borges , E. Buisson , M. G. G. Camargo , L. F. Cancian , et al. 2016. “Linking Plant Phenology to Conservation Biology.” Biological Conservation 195: 60–72. 10.1016/j.biocon.2015.12.033.

[eap3070-bib-0034] Nevill, P. G. , S. Tomlinson , C. P. Elliott , E. K. Espeland , K. W. Dixon , and D. J. Merritt . 2016. “Seed Production Areas for the Global Restoration Challenge.” Ecology and Evolution 6: 7490–7497. 10.1002/ece3.2455. 28725415 PMC5513262

[eap3070-bib-0035] Orme, D. , R. Freckleton , G. Thomas , T. Petzoldt , S. Fritz , N. Isaac , and W. Pearse . 2018. “caper: Comparative Analyses of Phylogenetics and Evolution in R.” R Package Version 1.0.1. https://cran.r-project.org/web/packages/caper/vignettes/caper.pdf.

[eap3070-bib-0036] R Core Team . 2020. R: A Language and Environment for Statistical Computing. Vienna: R Foundation for Statistical Computing. https://www.R-project.org/.

[eap3070-bib-0037] Ramírez‐Soto, A. , B. Villa‐Bonilla , C. R. Lucio‐Palacio , L. L. Libreros , L. R. Sánchez‐Velásquez , and E. R. Inzunza . 2018. “Mexico's Official Reforestation Programs Are Shrinking and Narrowing its Vision at a Higher Financial Expense.” Forest Policy and Economics 94: 32–34. 10.1016/j.forpol.2018.06.006.

[eap3070-bib-0038] Reed, P. B. 1988. National List of Plant Species that Occur in Wetlands. Report No. 88(26.3). Washington DC: U.S. Fish and Wildlife Service.

[eap3070-bib-0039] Rodrigues, R. R. , R. A. F. Lima , S. Gandolfi , and A. G. Nave . 2009. “On the Restoration of High Diversity Forests: 30 Years of Experience in the Brazilian Atlantic Forest.” Biological Conservation 142: 1242–1251. 10.1016/j.biocon.2008.12.008.

[eap3070-bib-0040] Sanderson, E. W. , M. Jaiteh , M. A. Levy , K. H. Redford , A. V. Wannebo , and G. Woolmer . 2002. “The Human Footprint and the Last of the Wild.” BioScience 52: 891–904. 10.1641/0006-3568(2002)052[0891:THFATL]2.0.CO;2.

[eap3070-bib-0041] Schwartz, M. W. , ed. 1997. Conservation in Highly Fragmented Landscapes. Boston, MA: Chapman & Hall.

[eap3070-bib-0042] Smith, S. A. , and J. W. Brown . 2018. “Constructing a Broadly Inclusive Seed Plant Phylogeny.” American Journal of Botany 105: 302–314. 10.1002/ajb2.1019. 29746720

[eap3070-bib-0043] Spyreas, G. 2019. “Floristic Quality Assessment: A Critique, a Defense, and a Primer.” Ecosphere 10: e02825. 10.1002/ecs2.2825.

[eap3070-bib-0044] Srivastava, D. S. , M. W. Cadotte , A. A. M. MacDonald , R. G. Marushia , and N. Mirotchnick . 2012. “Phylogenetic Diversity and the Functioning of Ecosystems.” Ecology Letters 15: 637–648. 10.1111/j.1461-0248.2012.01795.x. 22583836

[eap3070-bib-0045] Swink, F. , and G. S. Wilhelm . 1979. Plants of the Chicago Region, 3rd ed. Lisle, IL: The Morton Arboretum.

[eap3070-bib-0046] Tallamy, D. W. , D. L. Narango , and A. B. Mitchell . 2021. “Do Non‐Native Plants Contribute to Insect Declines?” Ecological Entomology 46: 729–742. 10.1111/een.12973.

[eap3070-bib-0047] Těšitel, J. , A. Li , K. Knotková , R. McLellan , P. C. G. Bandaranayake , and D. W. Watson . 2021. “The Bright Side of Parasitic Plants: What Are They Good for?” Plant Physiology 185: 1309–1324. 10.1093/plphys/kiaa069. 33793868 PMC8133642

[eap3070-bib-0048] The Plant List . 2010. “The Plant List.” Version 1. http://www.theplantlist.org/.

[eap3070-bib-0049] Tillman, S. C. , and J. W. Matthews . 2023. “Evaluating the Ability of Wetland Mitigation Banks to Replace Plant Species Lost from Destroyed Wetlands.” Journal of Applied Ecology 60: 990–998. 10.1111/1365-2664.14391.

[eap3070-bib-0050] University of Minnesota . 2020. Plant Information Online. Minneapolis, MN: University of Minnesota. https://www.plantinfo.org/.

[eap3070-bib-0051] US Army Corps of Engineers . 2020. “NWPL – National Wetland Plant List.” https://wetland-plants.sec.usace.army.mil/.

[eap3070-bib-0052] USDA NRCS . 2020. The PLANTS Database. Greensboro, NC: National Plant Data Team. http://plants.usda.gov/.

[eap3070-bib-0053] Van Kleunen, M. , F. Essl , J. Pergl , G. Brundu , M. Carboni , S. Dullinger , R. Early , et al. 2018. “The Changing Role of Ornamental Horticulture in Alien Plant Invasions.” Biological Reviews 93: 1421–1437. 10.1111/brv.12402. 29504240

[eap3070-bib-0054] Vidal, C. Y. , R. P. Naves , R. A. G. Viani , and R. R. Rodrigues . 2020. “Assessment of the Nursery Species Pool for Restoring Landscapes in Southeastern Brazil.” Restoration Ecology 28: 427–434. 10.1111/rec.13096.

[eap3070-bib-0055] Violle, C. , W. Thuiller , N. Mouquet , F. Munoz , N. J. B. Kraft , M. W. Cadotte , S. W. Livingstone , and D. Mouillot . 2017. “Functional Rarity: The Ecology of Outliers.” Trends in Ecology & Evolution 32: 356–367. 10.1016/j.tree.2017.02.002. 28389103 PMC5489079

[eap3070-bib-0056] White, A. , J. B. Fant , K. Havens , M. Skinner , and A. T. Kramer . 2018. “Restoring Species Diversity: Assessing Capacity in the U.S. Native Plant Industry.” Restoration Ecology 26: 605–611. 10.1111/rec.12705.

[eap3070-bib-0057] White, A. S. 2016. “From Nursery to Nature: Evaluating Native Herbaceous Flowering Plants Versus Native Cultivars for Pollinator Habitat Restoration.” Graduate Dissertation. The University of Vermont.

[eap3070-bib-0058] Wilhelm, G. , and L. Rericha . 2017. Flora of the Chicago Region: A Floristic and Ecological Synthesis. Indianapolis, IN: Indiana Academy of Science.

[eap3070-bib-0059] Zanne, A. E. , D. C. Tank , W. K. Cornwell , J. M. Eastman , S. A. Smith , R. G. FitzJohn , D. J. McGlinn , et al. 2014. “Three Keys to the Radiation of Angiosperms into Freezing Environments.” Nature 506: 89–92. 10.1038/nature12872. 24362564

[eap3070-bib-0061] Zinnen, J. , R. Barak , and J. Matthews . 2024. Data for Influence of Ecological Characteristics and Phylogeny on Native Plant species' Commercial Availability. Urbana, IL: University of Illinois at Urbana‐Champaign. 10.13012/B2IDB-1143125_V1. PMC11725693 39691952

[eap3070-bib-0062] Zinnen, J. , L. M. Broadhurst , P. Gibson‐Roy , T. A. Jones , and J. W. Matthews . 2021. “Seed Production Areas Are Crucial to Conservation Outcomes: Benefits and Risks of an Emerging Restoration Tool.” Biodiversity and Conservation 30: 1233–1256. 10.1007/s10531-021-02149-z.

[eap3070-bib-0063] Zinnen, J. , and J. W. Matthews . 2022. “Native Species Richness of Commercial Plant Vendors in the Midwestern United States.” Native Plants Journal 23: 4–16. 10.3368/npj.23.1.4.

[eap3070-bib-0064] Zinnen, J. , G. Spyreas , D. N. Zaya , and J. W. Matthews . 2021. “Niche Ecology in Floristic Quality Assessment: Are Species with Higher Conservatism more Specialized?” Ecological Indicators 121: 107078. 10.1016/j.ecolind.2020.107078.

