# Supplementary material for: Influence of ecological characteristics and phylogeny on native plant species' commercial availability
Source: Ecol Appl. 2024 Dec 18;35(1):e3070. doi: 10.1002/eap.3070 (PMC11725693; doi:10.1002/eap.3070)
Supplement: Supplementary file 1 — Appendix S1: [file EAP-35-e3070-s001.pdf]

Appendix S1

Jack Zinnen, Rebecca S. Barak, and Jeffrey W. Matthews. Influence of ecological characteristics and phylogeny on native plant species' commercial availability. *Ecological Applications*.

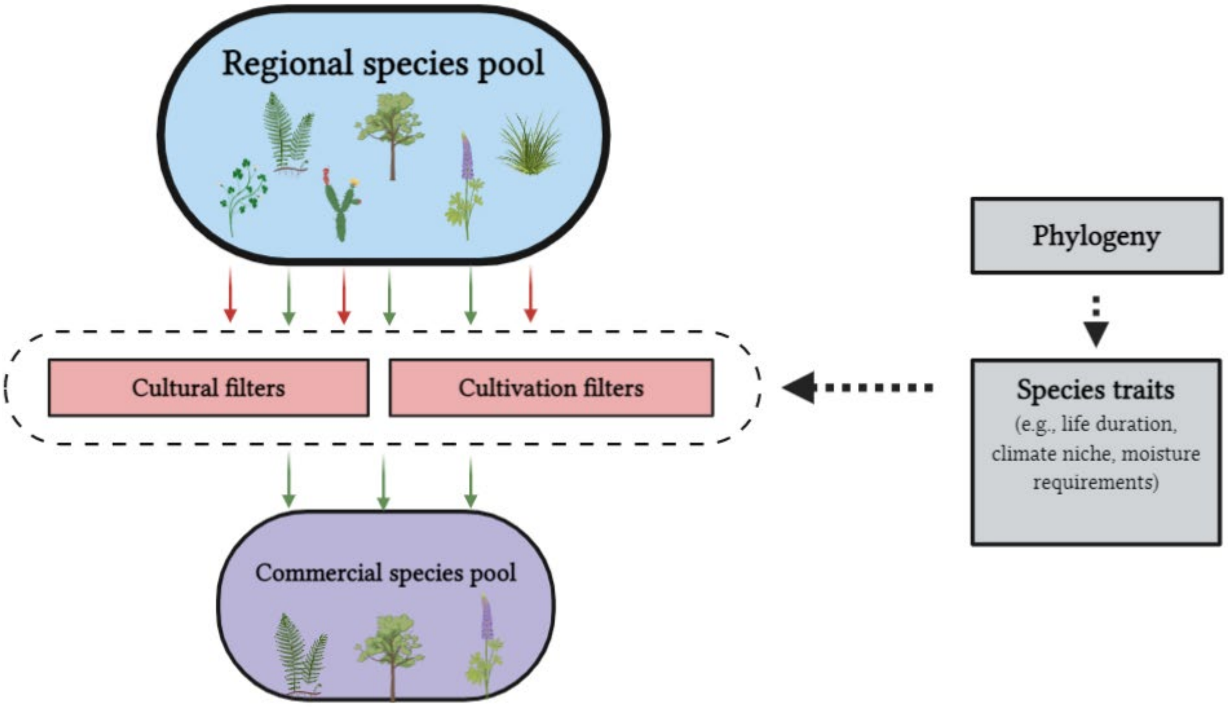

Figure S1: A conceptualized species pool representation for commercial availability. In a species pool conceptual model, a larger regional pool of species is gradually removed by a series of filters. In this case, the regional species pool is filtered by "cultural filters," or unattractiveness (e.g., weedy, obscure), as well as cultivation filters (e.g., unamenable to cultivation due to niche specialization). Dotted arrows represent an effect, whereas solid arrows reflect species that are interacting with the filters. Red arrows represent when a species does not achieve presence in the commercial species pool due to its traits. Species traits, which are linked to phylogeny, can affect species' probability of inclusion in the commercial pool. See Aronson et al. (2016), Pearse et al. (2018), and Cavender-Bares et al. (2020) for similar species pool diagrams in relation to the commercial trade.

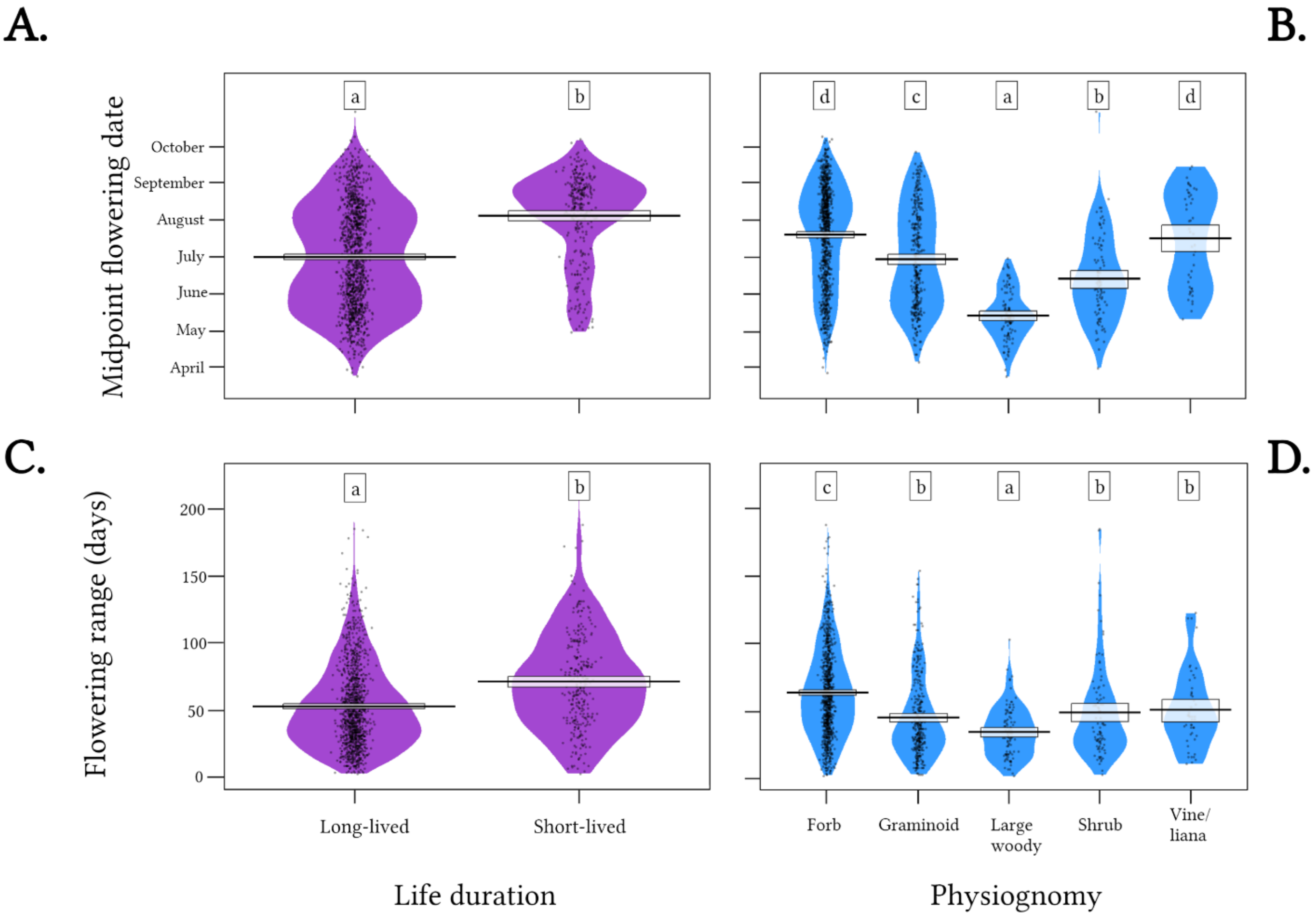

Figure S2: Violin plots showing that lifespan (A; C) and growth form (B; D) are associated with significant differences in midpoint blooming date and blooming duration (i.e., "range"). Uncapitalized letters represent significant differences at α = 0.05 for Wilcoxon rank-sum (subpanels A and C) following Kruskal–Wallis tests (subpanels B and D) showing significant differences across the multiple growth forms.

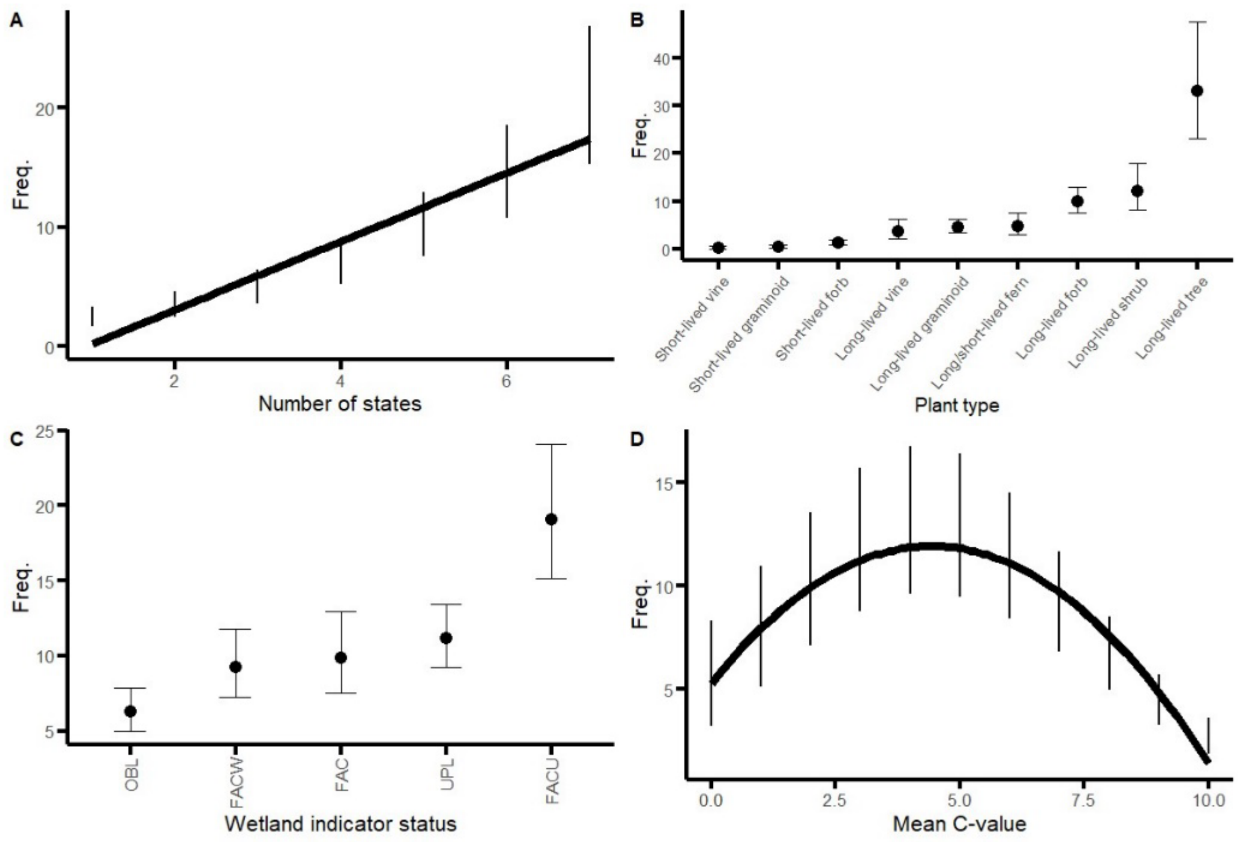

Figure S3: Commercial frequency is associated with four ecological factors. Frequency for each factor was shown as estimated marginal mean counts using *ggeffects* (Lüdecke 2018); shown marginal means are from the best supported model that included all four ecological factors, and a quadratic mean C-value term, in a negative binomial GLM. Frequency was greater for species with a greater number of native inhabited states (A), and woody plant types (B). Frequency was greater for facultative upland species (C), and those with intermediate mean coefficients of conservatism (C-value; D). For subpanel C, UPL = upland, FACU = facultative upland, FAC = facultative, FACW = facultative wetland, and OBL = obligate wetland. For subpanels A and D, vertical black lines show the 95% confidence interval for the estimated proportions per integer. Dots in subpanels B and C represent the marginal mean counts of frequency, and lines with bars indicate 95% confidence intervals.

Table S1: Comparisons of models that explain commercial availability (presence/absence) and frequency. Availability models were binomial GLMs; frequency models were negative binomial models. All model iterations are shown in this table. For these analyses, 79 species were excluded because they did not have C-values; included are 2,840 species from the total species pool. Model terms: A = availability; F = frequency; S = state count; P = plant type; W = wetland indicator status; C = mean C-value.

	**Model**	**df**	**AICc**	**ΔAICc**	**weight**
Availability	$A \sim S + P + W + C + C^2$	16	3039.1	0.0	0.991
	$A \sim S + P + C + C^2$	12	3048.6	9.5	0.009
	$A \sim S + P + W + C^2$	15	3065.2	26.1	< 0.001
	$A \sim S + P + C^2$	11	3072.4	33.3	< 0.001
	$A \sim S + P + W + C$	15	3075.8	36.7	< 0.001
	$A \sim S + P + W$	14	3080.5	41.4	< 0.001
	$A \sim S + P + C$	11	3086	46.8	< 0.001
	$A \sim S + P$	10	3098.3	59.2	< 0.001
	$A \sim P + W + C + C^2$	15	3354.3	315.2	< 0.001
	$A \sim S + W + C + C^2$	8	3356.3	317.1	< 0.001
	$A \sim P + C + C^2$	11	3367.7	328.5	< 0.001
	$A \sim S + C + C^2$	4	3376.6	337.5	< 0.001
	$A \sim P + W + C^2$	14	3405.4	366.3	< 0.001
	$A \sim P + C^2$	10	3415.1	375.9	< 0.001
	$A \sim S + W + C^2$	7	3443.3	404.2	< 0.001
	$A \sim S + W$	6	3445.2	406.1	< 0.001
	$A \sim S + W + C$	7	3446.9	407.8	< 0.001
	$A \sim P + W + C$	14	3457.1	418	< 0.001
	$A \sim S + C^2$	3	3458.7	419.6	< 0.001
	$A \sim S$	2	3468.4	429.2	< 0.001
	$A \sim P + C$	10	3468.7	429.6	< 0.001
	$A \sim S + C$	3	3469.4	430.2	< 0.001
	$A \sim P + W$	13	3566.7	527.6	< 0.001
	$A \sim P$	9	3603.1	564	< 0.001
	$A \sim W + C + C^2$	7	3647.4	608.3	< 0.001
	$A \sim C + C^2$	3	3667.8	628.7	< 0.001
	$A \sim W + C^2$	6	3775.5	736.3	< 0.001
	$A \sim C^2$	2	3788.8	749.7	< 0.001
	$A \sim W + C$	6	3825.5	786.4	< 0.001
	$A \sim C$	2	3844.3	805.2	< 0.001
	$A \sim W$	5	3867.2	828.1	< 0.001
	$A \sim 1$	1	3906.5	867.4	< 0.001
Frequency	$F \sim S + P + W + C + CC^2$	17	12470.9	0.0	> 0.999
	$F \sim S + P + W + CC^2$	16	12494.2	23.3	< 0.001
	$F \sim S + P + W + C$	16	12517.3	46.4	< 0.001
	$F \sim S + P + C + CC^2$	13	12526.3	55.4	< 0.001
	$F \sim S + P + CC^2$	12	12538.5	67.5	< 0.001
	$F \sim S + P + C$	12	12559	88.1	< 0.001

F ~ S + P + W	15	12563.1	92.2	< 0.001
F ~ S + P	11	12619	148.1	< 0.001
F ~ P + W + C + CC^2	16	12678	207.1	< 0.001
F ~ P + C + CC^2	12	12733	262	< 0.001
F ~ P + W + CC^2	15	12738.5	267.5	< 0.001
F ~ P + CC^2	11	12775.1	304.2	< 0.001
F ~ P + W + C	15	12797.5	326.5	< 0.001
F ~ S + W + C + CC^2	9	12822.8	351.9	< 0.001
F ~ P + C	11	12830.8	359.9	< 0.001
F ~ S + W + CC^2	8	12887.6	416.7	< 0.001
F ~ S + C + CC^2	5	12899.8	428.8	< 0.001
F ~ S + W + C	8	12903	432.1	< 0.001
F ~ S + W	7	12907.7	436.7	< 0.001
F ~ P + W	14	12931.7	460.7	< 0.001
F ~ S + CC^2	4	12944.2	473.3	< 0.001
F ~ S + C	4	12962.4	491.5	< 0.001
F ~ W + C + CC^2	8	12969	498.1	< 0.001
F ~ S	3	12976.7	505.8	< 0.001
F ~ P	10	12994.3	523.3	< 0.001
F ~ C + CC^2	4	13033.7	562.8	< 0.001
F ~ W + CC^2	7	13079.4	608.5	< 0.001
F ~ CC^2	3	13123.1	652.2	< 0.001
F ~ W + C	7	13129.1	658.1	< 0.001
F ~ C	3	13174.7	703.8	< 0.001
F ~ W	6	13187.1	716.2	< 0.001
F ~ 1	2	13254.1	783.2	< 0.001

Table S2: Comparisons of binomial linear mixed effects models that predict availability based on two phenological traits, blooming phenology range and midpoint blooming date. A random effect of plant type was added to account for growth form and lifespan having significant effects on these characteristics (Appendix 1: Figure S2). Model terms: A = availability; D = blooming phenology range (days); M = midpoint blooming date (day number of year); P = plant type; | designates a random effect.

	**Model**	**df**	**AICc**	**ΔAICc**	**weight**
Blooming phenology range	A ~ D + (D | P)	5	1774.7	0.0	0.778
	A ~ 1 + (D | P)	4	1777.2	2.5	0.222
	A ~ 1 + (1 | P)	2	1790.8	16.1	< 0.001
Midpoint blooming date	A ~ 1 + (M | P)	4	1788.9	0.0	0.549
	A ~ M + (M | P)	5	1790.5	1.7	0.238
	A ~ 1 + (1 | P)	2	1790.8	1.9	0.231

Table S3: Parasitic genera used in a supplemental analysis to test whether parasitic species were less likely to be available than species in the rest of the data set. This hypothesis was tested using a one-way binomial exact test. For simplicity, some families (namely, Orchidaceae and fern families) that are often parasites during their early life cycle were not included.

**Family** (with genera)	Number available / species richness
**Convolvulaceae**	0 / 12
Cuscuta	
**Ericaceae**	0 / 3
Pterospora, *Monotropa*	
**Orobanchaceae**	12 / 33
Agalinis, *Aureolaria*, *Buchnera*, *Castilleja*, *Dasistoma*, *Epifagus*, *Euphrasia*, *Melampyrum*, *Orobanche*, *Pedicularis*	
**Santalaceae**	1 / 4
Arceuthobium, *Comandra*, *Geocaulon*, *Phoradendron*	

References

Aronson, M. F. J., C. H. Nilon, C. A. Lepczyk, T. S. Parker, P. S. Warren, S. S. Cilliers, M. A. Goddard, A. K. Hahs, C. Herzog, M. Katti, F. A. La Sorte, N. S. G. Williams, and W. Zipperer. 2016. "Hierarchical filters determine community assembly of urban species pools." *Ecology* 97: 2952–63. https://doi.org/10.1002/ecy.1535

Cavender-Bares, J., J. J. Padullés Cubino, W. D. Pearse, S. E. Hobbie, A. J. Lange, S. Knapp, and K. C. Nelson. 2020. "Horticultural availability and homeowner preferences drive plant diversity and composition in urban yards." *Ecological Applications* 30: e202082. https://doi.org/10.1002/eap.2082

Lüdecke, D. 2018. "Ggeffects: Tidy Data Frames of Marginal Effects from Regression Models." *Journal of Open Source Software* 3: 772.

Pearse, W. D., J. Cavender-Bares, S. E. Hobbie, M. L. Avolio, N. Bettez, R. Roy Chowdhury, L. E. Darling, P. M. Groffman, J. M. Grove, S. J. Hall, J. B. Heffernan, J. Learned, C. Neill, K. C. Nelson, D. E. Pataki, B. L. Ruddell, M. K. Steele, and T. L. E. Trammell. 2018. "Homogenization of plant diversity, composition, and structure in North American urban yards." *Ecosphere* 9: e02105. https://doi.org/10.1002/ecs2.2105
